# Burst Firing and Spatial Coding in Subicular Principal Cells

**DOI:** 10.1523/JNEUROSCI.1656-18.2019

**Published:** 2019-05-08

**Authors:** Jean Simonnet, Michael Brecht

**Affiliations:** ^1^Bernstein Center for Computational Neuroscience Berlin, Humboldt-Universität zu Berlin, 10115 Berlin, Germany, and; ^2^NeuroCure Cluster of Excellence, Humboldt-Universität zu Berlin, 10099 Berlin, Germany

**Keywords:** border cell, cluster analysis, hippocampus, multiplexing, orientation

## Abstract

The subiculum is the major output structure of the hippocampal formation and is involved in learning and memory as well as in spatial navigation. Little is known about how neuronal diversity contributes to function in the subiculum. Previously, *in vitro* studies have identified distinct bursting patterns in the subiculum. Here, we asked how burst firing is related to spatial coding *in vivo*. Using juxtacellular recordings in freely moving male rats, we studied the bursting behavior of 102 subicular principal neurons and distinguished two populations: sparsely bursting (∼80%) and dominantly bursting neurons (∼20%). These bursting behaviors were not linked to anatomy: both cell types were found all along the proximodistal and radial axes of the subiculum and all identified cells were pyramidal neurons. However, the distinct burst firing patterns were related to functional differences: the activity of sparsely bursting cells showed a stronger spatial modulation than the activity of dominantly bursting neurons. In addition, all cells classified as boundary cells were sparsely bursting cells. In most sparsely bursting cells, bursts defined sharper firing fields and carried more spatial information than isolated spikes. We conclude that burst firing is functionally relevant to subicular spatially tuned neurons, possibly by serving as a mechanism to transmit spatial information to downstream structures.

**SIGNIFICANCE STATEMENT** The subiculum is the major output structure of the hippocampal formation and is involved in spatial navigation. *In vitro*, subicular cells can be distinguished by their ability to initiate bursts as brief sequences of spikes fired at high frequencies. Little is known about the relationship between cellular diversity and spatial coding in the subiculum. We performed high-resolution juxtacellular recordings in freely moving rats and found that bursting behavior predicts functional differences between subicular neurons. Specifically, sparsely bursting cells have lower firing rates and carry more spatial information than dominantly bursting cells. Additionally, bursts fired by sparsely bursting cells encoded spatial information better than isolated spikes, indicating that bursts act as a unit of information dedicated to spatial coding.

## Introduction

The subiculum is the major output structure of the hippocampus, receiving its main inputs from CA1 and sending divergent outputs to many subcortical and cortical areas (Amaral and Witter, 1989; Witter, 2006). The subiculum is involved in spatial learning and memory (Morris et al., 1990; Galani et al., 1998; Roy et al., 2017; Cembrowski et al., 2018) but has not been the major focus of studies analyzing hippocampal function in spatial navigation.

*In vivo*, the vast majority of subicular neurons carry positional information (Sharp and Green, 1994; Brotons-Mas et al., 2010, 2017) but subicular firing fields are not as well defined as those of CA1 place cells or the eye-catching medial entorhinal grid cells (O'Keefe and Dostrovsky, 1971; Hafting et al., 2005), because of higher basal firing rates in the subiculum compared with cells from these two other areas. Nevertheless, the subiculum spatial signals could play a determining role in forming and stabilizing other spatial codes. Subicular neurons seem to generalize across different environments because firing fields of subicular cells do not remap in response to novel environments, nor do they remap in darkness (Sharp, 1997; Lever et al., 2009; Brotons-Mas et al., 2010). The encoding of environmental boundaries, via boundary cells, is a prominent feature of the subiculum (Barry et al., 2006; Lever et al., 2009; Lee et al., 2018), which is likely to play a role in forming and anchoring spatial maps in adult animals (O'Keefe and Burgess, 1996; Hartley et al., 2000; Barry et al., 2006; Hardcastle et al., 2015; Krupic et al., 2015) and from early stages of development (Bjerknes et al., 2014; Muessig et al., 2015).

Determining how the subiculum generates its own activity and could influence spatial signals in other areas requires further investigation, more specifically at the level of the microcircuit. Indeed, the microcircuitry underlying spatial tuning in the subiculum is largely unresolved. The subicular anatomy is not as clearly stratified as the stratum pyramidale of CA1 (proximal to subiculum) and also lacks the elaborate lamination of six-layered cortical structures such as the presubiculum (distal to subiculum). The analysis of cell morphology indicates some internal structure (O'Mara, 2005) as well as laminar or modular organization based on long-range connectivity (Naber and Witter, 1998; Ishizuka, 2001; Witter, 2006; Y. Kim and Spruston, 2012). *In vitro*, subicular principal neurons may be distinguished by their firing patterns: some are intrinsic bursting (from 45 to 80%) and others are regular spiking cells (Greene and Totterdell, 1997; Staff et al., 2000). Bursting relates to subicular anatomy; deeper cells as well as cells located on the distal part are more likely to be intrinsic bursting neurons (Greene and Totterdell, 1997; E. Harris et al., 2001; Y. Kim and Spruston, 2012; Cembrowski et al., 2018). However, how bursting relates to subicular function remains mostly unresolved, even though a few functional correlates of bursting have been suggested. First, the biophysical properties of subicular cells could be predicted by their efferent target area (Y. Kim and Spruston, 2012; Cembrowski et al., 2018), suggesting that intrinsic bursting or regular spiking cells might generate different streams of information (Cembrowski et al., 2018). Lastly, local connectivity and recruitment by sharp wave ripples suggested distinct roles for regular spiking and intrinsic bursting cells in the subicular microcircuit function (Böhm et al., 2015).

Here, we asked how subicular bursting relates to spatial coding *in vivo*. We took advantage of high-resolution juxtacellular recordings, which enabled us to reliably resolve small amplitude spikes; especially those resulting from sodium-channel inactivation during bursts. Using this technique in freely moving rats we asked: (1) Can bursting patterns be used to classify subicular neurons *in vivo* as *in vitro*? (2) How does the burstiness of discharges relate to spatial coding? (3) Do bursts and isolated spikes convey different types of information? We classified cells based on their burstiness and found that sparsely bursting cells are more spatially modulated than dominantly bursting cells. In addition, we found that all subicular boundary cells were sparsely bursting cells. In a large fraction of spatially modulated neurons (34/51, 66%), we found that bursts encoded position significantly better than isolated spikes. Here, bursts are distinct units containing more spatial information than isolated spikes. Because bursts and isolated spikes are differently encoded by short-term synaptic dynamics, our results imply that spatial information should be transmitted more effectively by facilitating synapses to downstream areas.

## Materials and Methods

All experimental procedures were performed according to German guidelines on animal welfare.

### 

#### 

##### Juxtacellular recordings in freely moving rats.

Experimental procedures for obtaining juxtacellular recordings in freely moving rats were performed similarly to earlier publications (Tang et al., 2014). Recordings were made in 40 male Long–Evans rats (150–350 g) maintained in a 12 h light/dark phase and recorded during the dark phase. Surgical procedures were all performed under ketamine (80 mg/kg) and xylazine (10 mg/kg) anesthesia. Rats were implanted with a head-implant including a metal post for head-fixation, a placement of a miniaturized preamplifier coupled to two LEDs (red and blue), and a protection cap. To target the dorsal subiculum, a plastic ring was glued to the skull surface 5.7–6 mm posterior to bregma and 2.9–3.2 mm left to midline. The craniotomy and the positioning of the metal post for holding the miniaturized micromanipulator (Kleindiek Nanotechnik) were done either during the same surgery or in a subsequent surgery. After implantation, rats were allowed to recover and were habituated to head-fixation for 2–5 d. Rats were trained to forage for chocolate pellets in an open-field arena, a 70 × 70 × 50 cm (WDH) box with a white polarizing cue card on one of the walls, before and after implantation (3–7 d, multiple sessions of 15–20 min each per day).

For recordings, rats were head-fixed and the miniaturized micromanipulator and preamplifier were secured to the metal posts.

Glass pipettes with resistance 4–6 MΩ were filled with Ringer solution (*n* = 103/109) containing the following (in mm): 135 NaCl, 5.4 KCl, 5 HEPES, 1.8 CaCl_2_, and 1 MgCl_2_; or patch-clamping internal solution (*n* = 6/109) containing the following (in mm): 130 K-gluconate, 10 Na-gluconate, 10 HEPES, 10 phosphocreatine, 4 MgATP, 0.3 GTP, 4 NaCl. In both cases, the pH was adjusted to 7.2, neurobiotin (1–2%) was added to the solution, and the osmolality was adjusted to 285–305 mmol/kg. The patch-clamp solution was used to perform juxtacellular stimulations, of which the results are not used in the context of the current study. The firing rate and the firing pattern were not different between subicular cells recorded with the two different solutions; therefore, the two subsets have been merged and considered as one group.

The glass recording pipette was advanced into the brain; and a thick agarose solution (3–4% in Ringer) was applied into the recording chamber for sealing the craniotomy and for stabilization. Animals were then released into the behavioral arena and juxtacellular recordings were established while animals were freely exploring the environment. The juxtacellular signals were acquired with an ELC-03XS amplifier (NPI electronic) and digitized with a Power 1401 data-acquisition interface coupled to Spike2-v7 (CED, Cambridge Electronic Design) where signals were sampled at 50 kHz. The arena was filmed from above with a color camera so the position of red and blue LEDs could be tracked to determine the animal's location and head-direction. All signal processing and analyses were performed in MATLAB (MathWorks).

##### Anatomy.

The neurobiotin in the pipette allowed us to perform juxtacellular labeling at the end of the recording session (Pinault, 1996; Tang et al., 2014). A number of recordings were either lost before the labeling could be attempted, or the recorded neurons could not be clearly identified, but the location of all the cells included in the current study was positively assigned to the subiculum. Ten to 30 min after the labeling protocol, the animals were killed by overdose of isoflurane, and perfused transcardially with 0.1 m PBS followed by 4% paraformaldehyde solution. Brains were dissected out of the animal's skull and were placed in the same 4% paraformaldehyde solution for 12–24 h, and then in 0.1 m PB. Parasagittal sections (60–150 μm thick) were obtained using a vibratome (Mikrom, HM 650 V, ThermoFisher Scientific). Sections were washed in PBS 0.1 m (2 × 10 min, agitation 60 rpm), in PBS 0.1 m containing 0.5% Triton (2 × 10 min, agitation 60 rpm), and then pre-incubated in PBS 0.1 m containing 2.5% BSA and 0.5% Triton (1 h at room temperature, agitation 60 rpm). Sections were then incubated with PBS 0.1 m containing 1:500 AlexaFluor488-streptavidin, 1% BSA and 0.5% Triton (overnight at 4°C, agitation 60 rpm), revealing the neurobiotin. Sections were then washed in PBS 0.1 m (2 × 10 min, agitation 60 rpm). Sections were not mounted, but were instead briefly transferred on slides for acquiring fluorescent signals (Leica DM 5500B) and then kept in PBS 0.1 m containing 0.01 m sodium azide at 4°C for short term storage (max 1–2 months).

We distinguished three levels along each one of the proximodistal and radial axes (depth) of the subiculum. From CA1, the first 2/5 was considered as proximal subiculum, the last 2/5 as distal subiculum, and the 1/5 in the middle as an intermediate part. We did not assign recordings from the most superficial 2/5 of the subiculum, mostly because it mainly contains fibers and interneurons. We defined the next three 1/5 as superficial, middle, and deep subiculum. Ideally, recovered cells or recording sites could be assigned to a proximodistal and depth level of the subiculum (*n* = 34/102). Only the proximodistal level of the recordings could be assigned using the pipette track location (*n* = 60/102). In some cases (*n* = 8/102), the pipette tracks had penetrated the subiculum following an angle that made the assignment impossible (e.g., proximal in the deeper part and distal in the most superficial part).

To reconstruct the morphology of recovered cells, we converted the fluorescent signals to a dark diaminobenzidine (DAB) precipitate so we could use a bright-field microscope (Olympus, BX 51) coupled with Neurolucida (MBF Bioscience) for reconstructing cellular morphologies. The conversion procedure was performed as follows: sections were washed in TBS (tris-HCl 0.05 m, 0.9% NaCl) (1 × 10 min, agitation 60 rpm) and then in TBS containing 0.3% Triton (TBS-X, 3 × 10 min); sections were then incubated with TBS-X containing 20% BSA for 20 min, quickly washed in TBS-X, and then incubated in the TBS-X containing 1:100 of the B solution of the Vectastain ABC-kit (Biozol; 4–6 h at room temperature, agitation 60 rpm). Sections were then incubated in TBS-X containing 1:100 of the A-B solutions (from the Vectastain ABC-kit) overnight at 4°C, then washed in TBS (1 × 10 min) and in PB 0.1 m (2 × 10 min); then, sections were incubated in a pre-staining solution composed of PB 0.1 m containing 0.004% NH_4_Cl, 0.2% glucose, 0.004% NH_4_NiSO_4_ and 5.5% 3,3′-DAB (20 min, darkness, agitation 60 rpm). The final step of the procedure was performed by incubating the sections with a staining solution (pre-staining solution + 2.4 U/ml of glucose oxidase) for 20–60 min at room temperature and then stopping the reaction by washing with PB 0.1 m (3 × 10 min). Sections were mounted in a non-aqueous medium (e.g., Eukitt, Sigma-Aldrich) to prevent fainting of Ni-precipitates.

##### Spike and bursts detection.

For spike detection, the raw signals were filtered (0.3–6 kHz, zero phase bandpass Butterworth filter of order 8). Transients were then detected using a threshold of 2.0 times the root mean square (rms) of the signal. High amplitude artifacts, because of behaviors like grooming, could increase the rms value significantly and prevent the detection of the smallest transients; the values in a window of 2.0 ms around these artifacts were therefore clipped and replaced by zeros. A second step for separating spikes from noise consisted of calculating the principal components of the transients followed by manually clustering the events into spikes and noise. This cleaning step was first performed on filtered waveforms and subsequently on raw waveforms. Eventually, the accuracy of spike detection was visually checked, scrolling throughout the whole recording. The cleaning step was repeated until the detection was optimal (minimizing false-positives and -negatives).

Finally, spikes were categorized as belonging to a burst if the interval from the prior spike and/or to the next spike was shorter than a threshold set at 6 ms. One burst was therefore defined as a group of spikes (≥2) interleaved with <6 ms. We calculated the proportion of bursts with >2 spikes as well as the mean spike count per burst as measures of burst strength. The burst time stamp was set to that of the first spike in a burst. The intraburst interval was set as the mean interspike interval (ISI) during bursts.

##### Spike waveform analysis.

The raw signals were filtered (6 kHz, zero phase low-pass Butterworth filter of order 8) to minimize high-frequency noise. Spike shape parameters were determined based on the spike average waveform calculated from these low-pass filtered traces. Before the calculation of the average spike, the single waveforms had to be properly aligned. To this end, every spike waveform was oversampled at 1000 kHz using a spline interpolation to better estimate its shape. Signal-to-noise ratios often differed between recordings and with it, the spike amplitude. To compare spike shape parameters between cells, the waveform was normalized so that the rising amplitude was 1 mV. We then calculated the derivative of each waveform. The threshold was set to the first sample, where both the voltage and its derivative were at least 5% of their maximal value. The rising amplitude (mV) was set to the difference of potential between the peak and the threshold voltage. The afterhyperpolarization was set to the point at minimum voltage after the peak, when the derivative ≥0. The spike duration was set to the threshold-to-afterhyperpolarization duration.

Spike accommodation is classically defined as a reduction in the amplitude of subsequent spikes fired within a burst. Here, it was calculated as the ratio of amplitudes (from peak to afterhyperpolarization) between the second and the first spikes of each burst. An accommodation of 1 means that there was no decrease of spike amplitude; 0.5 means that amplitude was divided by 2.

Putative fast-spiking interneurons (*n* = 6/109) were identified based on four criteria: their high firing rate (typically >30 Hz), their spike duration (typically to 0.6 ms), their high maximum derivative (typically >9 mV/ms), and low minimum derivative (typically <−9 mV/ms). Cells meeting at least three of these four criteria were considered as fast-spiking interneurons and were not used for the subsequent analyses.

##### Analysis of burstiness.

*In vivo*, firing pattern features and burstiness are commonly addressed using methods relying on either distribution of ISIs or spike autocorrelations.

The first set of methods, based on the distribution of ISIs, has previously been used to study burstiness in the subiculum (Sharp and Green, 1994; Lever et al., 2009; Brotons-Mas et al., 2010) and other areas such as parahippocampal cortices (Ebbesen et al., 2016). In principle, ISI distribution analyses estimate burstiness by either determining a bursting index, being the proportion of ISI below the bursting threshold (here we used 6 ms) or by classifying the cells after performing a principal component analysis of the ISI histogram matrix. The limit of these analyses is that ISI interval distribution might overestimate burstiness for cells with elevated firing rates, and therefore they report a variability that correlates with cells' firing rates (S. M. Kim et al., 2012).

The second set of methods rely on spike autocorrelations (S. M. Kim et al., 2012; Latuske et al., 2015; Coletta et al., 2018). Latuske et al. (2015), as well as Coletta et al. (2018) both used principal component analysis of the first 12 ms of the spike-autocorrelation to classify the cells in different bursting groups. S. M. Kim et al. (2012) calculated a bursting index as the ratio of the integrated power of the autocorrelogram between 1 and 6 ms normalized by the overall power between 1 and 20 ms. Using these methods does not bias the burstiness estimation for high firing neurons (S. M. Kim et al., 2012). However, we realized that it could overestimate the bursting probability of neurons with low firing rates and occasional bursts because only the first few milliseconds of the spike autocorrelation were considered.

**Figure 1.**
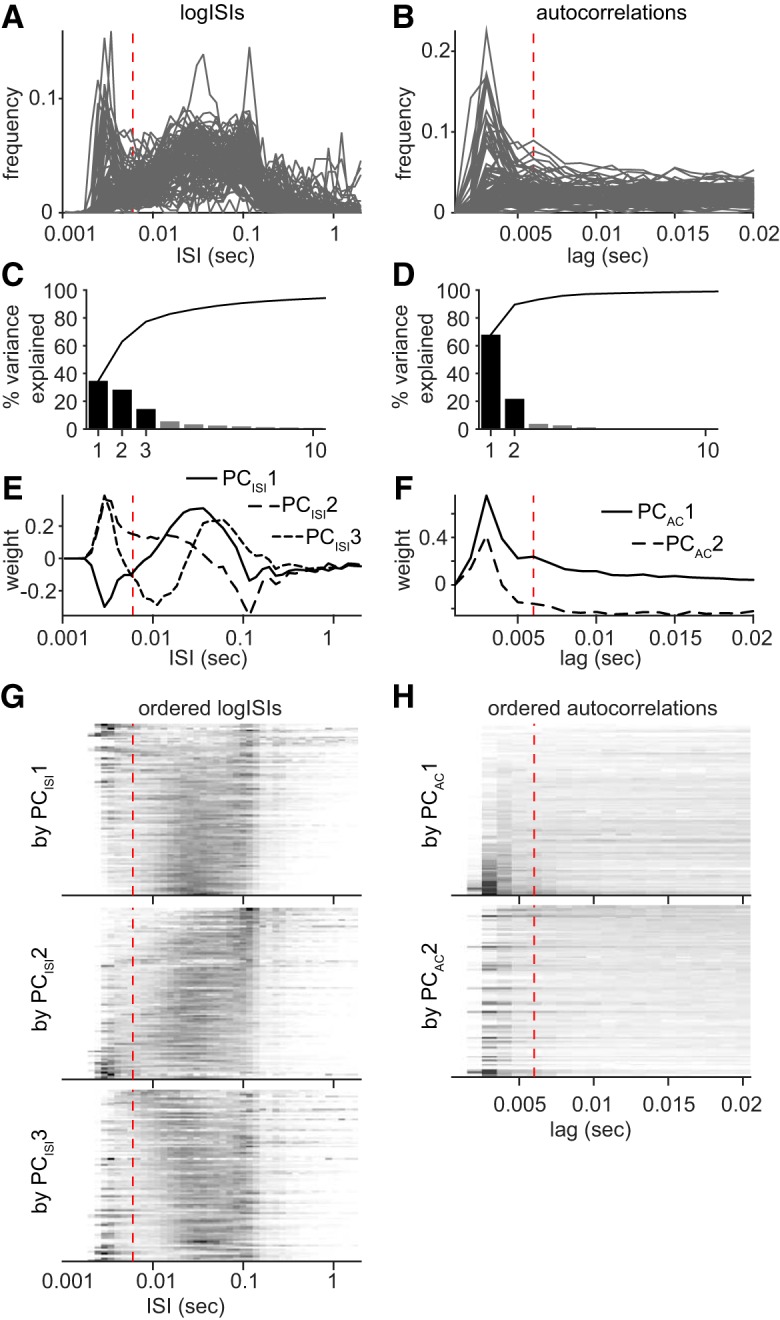
Principal component analyses of ISI distributions and spike autocorrelations during navigation. ***A***, Top, Histogram of the logISI for all subicular principal cells (*n* = 102). The values of the *x*-axis have been replaced by the corresponding interval values in seconds. ***B***, Top, Spike autocorrelation for all subicular principal cells (*n* = 102). The plot is normalized so the 0 ms-lag value is equal to 1 (out of the axis range here). The vertical dashed line is placed at 6 ms. Only navigating periods have been considered for these analyses (rat's speed >3 cm/s). ***C***, Percentage of variance explained by the 10 first principal components of logISI histogram. PC_ISI_1, PC_ISI_2 and PC_ISI_3 explain, respectively, 35, 29, and 15% of the total variance, so approximately 78% in total. ***D***, Percentage of variance explained by the 10 first principal components of spike autocorrelations. PC_AC_1 and PC_AC_2 explain, respectively, 68 and 22% of the total variance, so 90% in total. ***E***, Loading of the logISI bins into the first three principal components (PC_ISI_1, PC_ISI_2 and PC_ISI_3) of the logISI histogram matrix. The vertical dashed line is placed at 6 ms corresponding to our threshold for burst firing. The short intervals (<6 ms) are similarly loaded in the first three components in contrast with the more delayed intervals. ***F***, The autocorrelation lags are loaded according to similar patterns into the first two principal components (PC_AC_1, and PC_AC_2). ***G***, The logISI histogram frequency matrix has been ordered according to values on PC_ISI_1, PC_ISI_2, and PC_ISI_3, and plotted in grayscale. One line is one cell; dark values correspond to high frequencies and light values to low frequencies. Cells with an initial peak tend to be distributed on one side (top or bottom), but not clearly grouped together. For each plot, a structure emerges in the delayed ISI range. Using the three first principal components of the logISI frequency matrix seems biologically relevant because they all depict both bursting and other discharge patterns, such as firing rates or theta modulation (peak ∼0.1 s). ***H***, The spike autocorrelation frequency matrix has been ordered according to values on PC_AC_1and PC_AC_2, and plotted in grayscale. One line is one cell; dark values correspond to high frequencies and light values to low frequencies. PC_AC_1 is clearly a good parameter to classify cells according to burstiness as cells with an initial peak (<6 ms) are grouped together at the bottom of the color plot. PC_AC_2 is not as good for predicting bursting behavior, even though a few cells with an initial peak can be found again at the bottom of the color plot.

**Figure 2.**
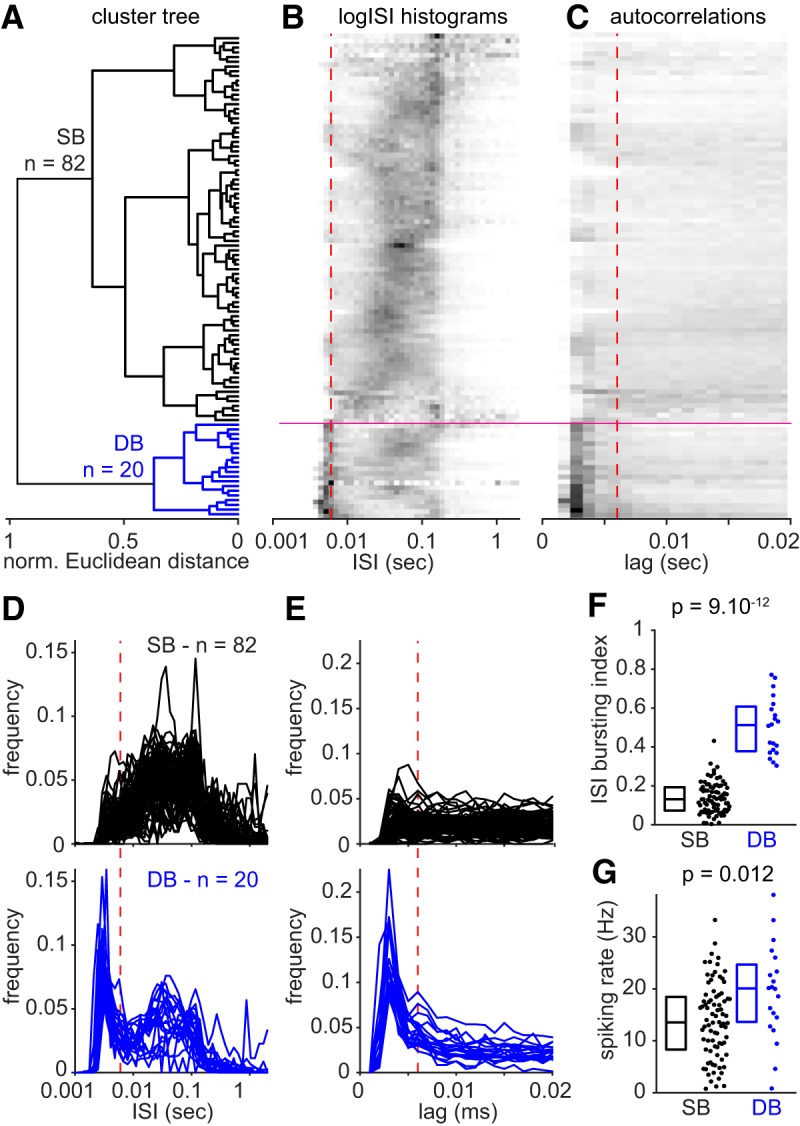
Classification of subicular principal cells based on their firing patterns during navigation. ***A***, Hierarchical cluster tree of subicular principal cells based on the logISI and spike autocorrelation principal component analyses. The Ward's method, an agglomerative hierarchical clustering procedure was used to generate the cluster tree based on normalized Euclidean distance between cells in a 5-dimensional space, defined by PC_ISI_1, PC_ISI_2, PC_ISI_3, PC_AC_1, and PC_AC_ 2. The black branch corresponds to SBs and the blue branch corresponds to DBs. ***B***, ***C***, logISI histogram and spike autocorrelation frequency matrices have been ordered as on the cluster tree in ***A*** and are represented in grayscale plots (black, high values; white, low values). The vertical dashed red lines are positioned at 6 ms. Horizontal magenta lines show the cluster separation on the color plots. Cells with a prominent initial peak in logISI histogram and spike autocorrelation are grouped at the bottom of the representations and correspond to dominantly bursting cells. ***D***, logISI histograms for SBs (*n* = 82; top) and DBs (*n* = 20; bottom). Note the prominent initial peak for dominantly bursting cells, absent for sparsely bursting cells, which highlights a higher proportion of low intervals corresponding to prominent burst firing. ***E***, Spike autocorrelations for sparsely bursting cells (*n* = 82, top) and dominantly bursting cells (*n* = 20, bottom). As for the logISI histogram, note the prominent initial peak for dominantly bursting cells, absent for sparsely bursting cells. ***F***, ISI-based bursting index corresponding to the proportion of ISIs <6 ms is significantly higher for dominantly bursting cells. ***G***, Spiking rate (Hz) is significantly higher for dominantly bursting cells. Statistics: two-tailed Mann–Whitney *U* test; box plots showing median and interquartile ranges.

Our classification of subicular principal cells in distinct subpopulations was inspired by the two different sets of methods. We used histograms of the logarithm of ISIs (logISI) instead of using only the initial bins of the ISI-histograms (e.g., 1–60 ms; Ebbesen et al., 2016). It has been used by others for burst analysis (Ko et al., 2012) and depicts the whole ISI distribution with a good focus on short intervals. Specifically, we defined 60 bins regularly spaced between log(0.0005) and log(10), so as to have a distribution of intervals between 0.0005 and 10 s. When logISI histograms were used in figures (Figs. 1, 2), the logISI *x*-axis values were replaced by the corresponding ISI values for more clarity. Principal component analysis was done on both the logISI frequency matrix and for the 1–20 ms lag frequency matrix (resolution of 1 ms). We focused on the first principal components (PCs) explaining most of the variance (Fig. 1*C*,*D*).

PC1, PC2 and PC3 of the logISI histograms (PC_ISI_1, PC_ISI_2, and PC_ISI_3) explained, respectively, 35, 29, and 15% of the total variance (approximately 78% total; Fig. 1*C*). PC1 and PC2 of the spike autocorrelation matrix (PC_AC_1 and PC_AC_2) explained 68 and 22% of the total variance (90% total; Fig. 1*D*). The loading of ISI and lags into these first components are represented in Figure 1, *E* and *F*, respectively. These first components from each PCA were used to represent the firing pattern features of all recorded neurons in a 5-dimensional space. We then generated a cluster tree using Ward's method on the normalized Euclidean distance between cells (Fig. 2*A*). The Ward's method establishes hierarchical clusters by iteratively grouping the two closest observations or groups of observations together. Consequently, cells with very similar firing patterns are primarily grouped together and groups with very different properties are linked at the end of the procedure (Ward, 1963). Two clusters strikingly emerged from the agglomerative cluster tree (Fig. 2*A*), defining two groups of neurons that we named sparsely bursting cells and dominantly bursting cells based on their potency to initiate bursts (see Results; Fig. 1). We compared our classification based on the first component of the spike autocorrelation only. The last resulted in a less relevant classification, where more groups should be defined to isolate the most bursting cells from the others (data not shown).

##### Analysis of spatial information.

The position of the rat was defined as the midpoint between the two head-mounted LEDs. A running speed threshold (3 cm/s) was applied for isolating periods of rest from navigation. For generating color-coded firing maps, space was discretized into pixels of 2.5 × 2.5 cm. For each such pixel the occupancy *o*(*x*) was calculated as follows:

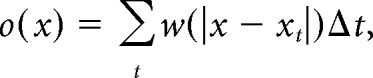
 where *x_t_* is the position of the rat at time *t*, Δ*t* the inter-frame interval, and *w* a Gaussian smoothing kernel with σ = 5 cm. Then, the firing rate *r* was calculated for each pixel *x*:

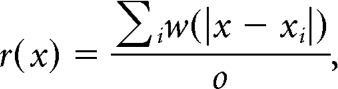
 where *x_i_* is the position of the rat when spike *i* was fired.

For recordings in which the animal's trajectory covered at least 60% of the open field (*n* = 84/102), we calculated the spatial information rate, *I* (bits/spike), from the spike train and rat trajectory as follows:

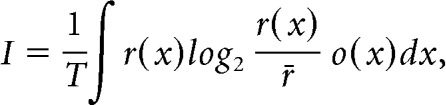
 where *r*(*x*) and *o*(*x*) are the firing rate and occupancy as a function of a given pixel *x* in the rate map. *r̄* is the overall mean firing rate of the cell, and *T* is the total duration of a recording session (Skaggs et al., 1993).

A cell was declared to have a significant amount of spatial information if the observed spatial information rate exceeded the 95th percentile of a random distribution of values of *I* obtained by circular shuffling. A circular time-shift *t′* ∈ [0 T] was applied to the recorded spike train timestamp (von Heimendahl et al., 2012; Bjerknes et al., 2014), *T* being the total duration of the recording session. This procedure maintains the temporal structure of the cell's firing but alters the spatial distribution of spikes and therefore *I*. It was performed 1000 times to generate the random distribution of *I* that was used to determine the significance of spatial information.

##### Analysis of boundary cells.

To determine whether subicular spatial neurons could be classified as border cells, we computed border scores (Solstad et al., 2008; Bjerknes et al., 2014) as follows. Firing fields were detected on the rate maps as a collection of neighboring pixels covering at least 100 cm^2^ (16 pixels) with a minimum rate of 1/3 of the firing rate range. Only fields with a peak firing of at least 2/3 of the firing rate range were considered. For each cell, we could detect the main and also secondary fields when they were present. For each detected field, the average rate (rate_field_) and the average distance from the closest wall (dist_field_), as well as the linear distance covered along the wall (cov_field_, average on the two first lines of pixels along the wall) were calculated. Each (bs_field_) was then calculated as follows:

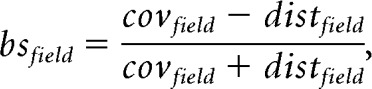
 bs_field_ values ranged from −1 to 1, −1 for fields that do not cover any wall, and 1 for fields that would perfectly line-up along one of the walls. We calculated each cell's border score (bs_cell_) by normalizing the contribution of each field *f* to their average rate:

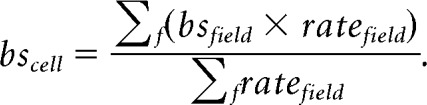
 Fields with higher rates have higher contribution on the bs_cell_. Border scores also ranged from −1 to 1 and are typically high for stereotypical boundary cells, with firing fields covering one or several boundaries. We tested the significance of border scores following the same procedure as for spatial information.

##### Statistical analyses.

We always show all data points on our population data plots. Boxplots always show medians and interquartile ranges.

In many cases, the significance of the difference observed between distinct groups was assessed with nonparametric tests only. Two-tailed Mann–Whitney *U* tests were used to determine whether two groups of unpaired observations were significantly different from each other (e.g., comparing sparsely bursting and dominantly bursting cells).

**Figure 4.**
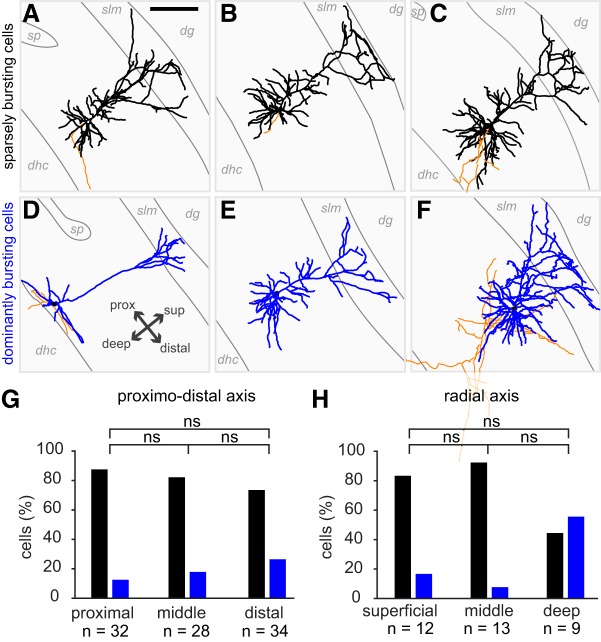
***A–F***, Reconstructions of 6 subicular principal cells. Dendrites are in black (sparsely bursting cells) or blue (dominantly bursting cells) and axons are in orange. Some of the anatomical outlines have been drawn, such as CA1 stratum pyramidale (sp), the stratum lacunosum moleculare (slm) and the limit of the subiculum with the dorsal hippocampal commissure (dhc). dg: dendate gyrus. All cells are oriented as indicated in panel ***D;*** prox: proximal; dist: distal; sup: superficial. In ***A***, scale bar = 200 μm. ***G,*** Distribution of sparsely bursting and dominantly bursting cells along the proximo-distal axis of the subiculum. Fisher's exact tests, with level of significance corrected to be equal 0.05 in total: ns: *p* > 0.05/3; proximal vs middle, *p* = .7211; middle vs distal = 0.5457; superficial vs distal = 0.2183. ***H,*** Distribution of sparsely bursting and dominantly bursting cells along the radial axis of the subiculum. Fisher's exact tests (ns: *p* > 0.05/3); superficial versus middle, *p* = 0.593; middle versus deep = 0.0231; superficial versus deep = 0.1588.

We used Fisher's exact tests to assess whether there was some significant trend within the distribution of cell types along the proximodistal and radial axes of the subiculum (Fig. 4*G*,*H*). Three levels were distinguished for each axis. We performed the Fisher's exact test on the three possible pairs (1–2, 2–3, 1–3) and corrected the *p* value for significance so the overall error α would be 0.05. The distribution within the two tested pairs would be considered different rather than random if the Fischer's exact test *p* value was < 0.0167 (0.05/3).

**Figure 5.**
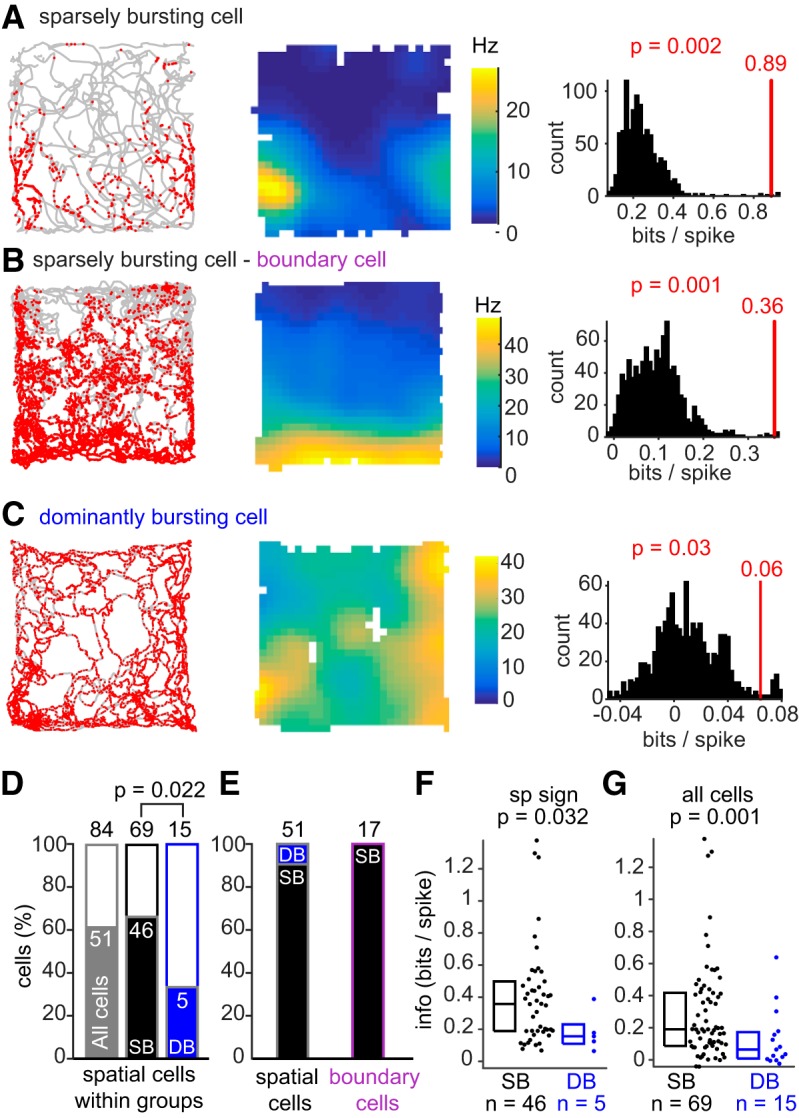
Sparsely bursting cells provide more spatial information than dominantly bursting cells. ***A***–***C***, Left, Trajectories of rat in gray with superimposed spikes in red. Middle, Corresponding rate maps with their color-map ranging from 0 to the maximum firing rate of the cells (Hz). Right, Cell-by-cell spatial significance analyses. Each cell's spatial information (red vertical line) was ranked within the distribution of spatial information values determined using a cell-by-cell circular shuffling procedure (black histogram, see Materials and Methods). For each cell, spatial information was declared significant if the cell's information exceeded the 95th percentile of the random distribution obtained after circular shuffling (see Materials and Methods). All the cells shown in ***A***–***C*** are spatially significant (*p* < 0.05; see Materials and Methods). Neurons in ***A*** and ***B*** are sparsely bursting cells; the neuron in ***C*** is a dominantly bursting cell. The neuron in ***B*** was categorized as a boundary cell (boundary score = 0.73, *p* = 0.01; see Materials and Methods). ***D***, Spatial information and significance were calculated for subicular cells recorded while the animal explored (speed >3 cm/s) at least 60% of the open-field arena (*n* = 84/102). The bar graph shows the percentage of spatially modulated cells from all subicular cells (*n* = 51/84; gray), and then from sparsely bursting cells (*n* = 46/69; black) and dominantly bursting cells (5/15 cells; blue). Sparsely bursting cells are more often spatially modulated than dominantly bursting cells (*p* = 0.022: significant Fisher's exact test). ***E***, Percentage of each cell type within spatial cell categories. Left, Sparsely bursting cells represent the majority (*n* = 46/51) of subicular spatial units. Right, All boundary cells were sparsely bursting cells ***F***, Spatial information calculated for spatially significant sparsely bursting and dominantly bursting cells (*p* = 0.032, bootstrapping, significant) ***G***, Spatial information calculated for all sparsely bursting cells and dominantly bursting cells (*p* = 0.001, bootstrapping, significant).

We used Fisher's exact tests to address whether spatially modulated cell types were equally distributed between the two populations of cells (Fig. 5*D*).

The size difference between the two clusters of subicular neurons and the small size of the group of dominantly bursting cells used for spatial information calculation [sparsely bursting cells (SB) = 48 vs dominantly bursting cells (DB) = 5] led us to test the significance of the difference with a bootstrapping procedure. *N* (number DB cells) values of spatial information were randomly selected from the sparsely bursting population. By repeating the procedure 1000 times, we could obtain a bootstrapped distribution of the median spatial information for sparsely bursting cells. The difference was significant if the rank of the median spatial information of dominantly bursting cells was within the fifth percentile of the bootstrapped distribution (*p* ≤ 0.05).

We wanted to test whether the spatial information encoded by bursts was significantly different from the spatial information encoded by isolated spikes. A direct comparison of spatial information values would not be appropriate, as the total number of events and smoothing parameters used for generating the rate maps can introduce bias in information measures (K. D. Harris et al., 2001). Consequently, we used a randomization method similar to K. D. Harris et al. (2001). In instances where there were less bursts than isolated spikes, we would compare information given by the *N* bursts to the information given by 1000 random subsets of *N* isolated spikes. Bursts were significantly more informative than isolated spikes if the rank of the burst spatial information exceeded the 95th percentile of the distribution of the spatial information given by the random subsets of isolated spikes.

In some instances where highly bursting cells had less isolated spikes than bursts (*n* = 3/102), we compared the information given by *N* isolated spikes to the information given by 1000 random subsets of *N* bursts. In this case, bursts were significantly more informative than isolated spikes if the rank of the isolated spike spatial information was within the fifth percentile of the distribution of the spatial information given by the random subsets of bursts.

## Results

We performed juxtacellular recordings in rats foraging for food in a 70 × 70 cm open-field arena. Our data consists of 102 subicular principal cells recorded in 40 rats. Neurons were assigned as subicular cells histologically and as principal cells based on firing rates and spike waveforms (see Materials and Methods).

### Sparsely and dominantly bursting cells: distinct firing patterns *in vivo*

Previous *in vitro* work (Greene and Totterdell, 1997; Staff et al., 2000; E. Harris et al., 2001; Y. Kim and Spruston, 2012) indicated the existence of distinct patterns of bursting in different types of subicular principal cells. In our *in vivo* recordings, we also noted distinct bursting patterns of subicular principal cells during navigation. We then categorized cells according to their burst discharge pattern during active locomotion (speed >3 cm/s).

First, we performed principal component analyses of histograms of the logarithm of ISI (logISI histograms) and spike autocorrelations (Fig. 1*A*,*B*). We focused on the first principal components (PC_ISI_ and PC_AC_) explaining most of the variance.

PC_ISI_1, PC_ISI_2, and PC_ISI_3 explained 35, 29, and 15% of the total variance (approximately 78% total; Fig. 1*C*). PC_AC_1 and PC_AC_2 explained 68 and 22% of the total variance (90% total; Fig. 1*D*). We checked how each component was describing cell's firing patterns. First, we looked at how each parameter (each logISI bins or autocorrelation lags) loaded onto each principal component (Fig. 1*E*,*F*). Second, we ordered the logISI histograms and autocorrelation matrices according the cells' value on each component and plotted them as grayscale images (Fig. 1*G*,*H*). To summarize, the selected five parameters successfully captured most features of subicular neurons' firing patterns, isolating bursting behavior as well as other temporal aspects of their firing.

Each subicular principal cell could therefore be positioned in a 5-dimensional space summarizing most of the firing pattern variability. From there an agglomerative cluster tree was generated based on the Euclidean distances between cells and groups of cells (Fig. 2*A*; see Materials and Methods for more detailed explanation) grouping cells according to their firing patterns features (Fig. 2*B*,*C*). Two distinct groups were identified: sparsely bursting cells (*n* = 82 of 102 cells, 80%) and dominantly bursting cells (*n* = 20 of 102 cells, ∼ 20%; Fig. 2*A–C*), named after their potential for burst initiation. Indeed, plotting each group separately showed that dominantly bursting cells displayed early peaks (<6 ms) in both logISI histograms (Fig. 2*D*) and spike autocorrelations (Fig. 2*E*) as opposed to sparsely bursting cells. The bursting index, calculated as the proportion of ISIs <6 ms, was significantly higher for dominantly bursting cells than for sparsely bursting cells (Fig. 2*F*; median: SB = 0.131, DB = 0.512, Mann–Whitney *U* test, *p* = 9.10^−12^). Dominantly bursting cells had higher bursting rates than sparsely bursting cells (medians: SB = 0.8 Hz, DB = 4.3 Hz; Mann–Whitney *U* test, *p* = 1.10^−8^). Firing rates were variable and rather high, as previously reported for subicular neurons (Sharp and Green, 1994; Lever et al., 2009; S. M. Kim et al., 2012). Firing rates were higher for dominantly bursting cells than for sparsely bursting cells (Fig. 2*G*; medians: SB = 13.6 Hz, DB = 20.1 Hz; Mann–Whitney *U* test, *p* = 0.012).

Having distinct burst indices, burst rates or firing rates between the two groups of cells was not unexpected, as our classification was established on firing pattern features. We then looked at the burst features and noticed further differences between sparsely bursting and dominantly bursting cells.

### Sparsely and dominantly bursting cells have distinct burst features

**Figure 3.**
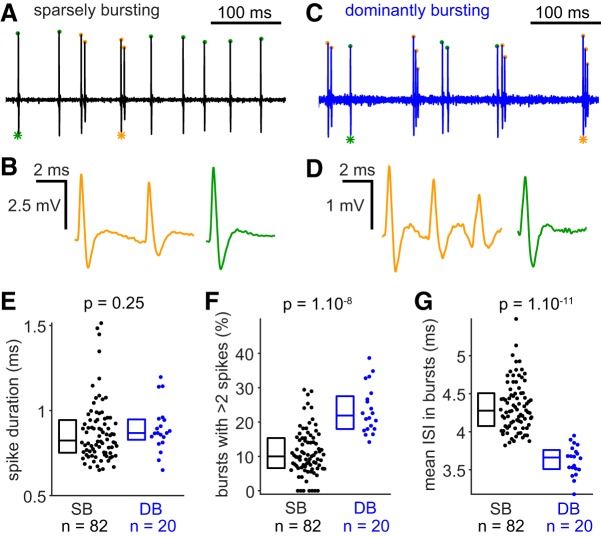
Spike and burst features of sparsely bursting cells and dominantly bursting cells. ***A***, Bandpass filtered (300–6000 Hz) trace of recording from a sparsely bursting cell. Spikes occurring in bursts are labeled with orange dots and isolated spikes with green dots. ***B***, Magnification of the burst (orange, left) and the isolated spike (green, right) indicated with a star. ***A*** and ***B*** have the same vertical scale. ***C***, Same as ***A*** for a dominantly bursting cell. ***D***, Same as ***B*** for a dominantly bursting cell. ***C*** and ***D*** have the same vertical scale. ***A*** and ***C***, as well as ***B*** and ***D*** have the same horizontal scale. ***E***, Spike duration, from threshold to afterhyperpolarization, is not different between sparsely bursting and dominantly bursting cells. ***F***, Bursts of dominantly bursting cells have more spikes than bursts fired by sparsely bursting cells. ***G***, The mean ISIs inside bursts are shorter in dominantly bursting cells compared with sparsely bursting cells. Statistics: two-tailed Mann–Whitney *U* test; box plots showing median and interquartile ranges.

Representative recordings and magnification of bursts and isolated spikes from sparsely bursting and dominantly bursting cells are shown in Figure 3*A–D*. Spike duration (from threshold to afterhyperpolarization) of sparsely bursting cells and dominantly bursting cells were not different from one another (Fig. 3*E*; median: SB = 0.824 ms, DB = 0.868 ms; Mann–Whitney *U* test, *p* = 0.25). In contrast, bursts seem to be intrinsically different between the two cells types (Fig. 3*B*,*D*). Bursts spike accommodation, quantified here as the ratio of the second spike amplitude to the first spike amplitude, is slightly more pronounced in dominantly bursting cells (median: SB = 0.817, DB = 777, Mann–Whitney *U* test, *p* = 0.009). The proportion of bursts with >2 spikes (Fig. 3*F*; median: SB = 10%, DB = 22%, Mann–Whitney *U* test, *p* = 1.10^−8^), as well as the average number of spikes per burst (median: SB = 2.1, DB = 2.27, Mann–Whitney *U* test, *p* = 1.10^−7^) was significantly higher in dominantly bursting cells. Finally, the intraburst intervals (ISI in bursts) are on average longer in sparsely bursting cells (Fig. 3*G*; median: SB = 4.29 ms, DB = 3.66 ms, Mann–Whitney *U* test, *p* = 1.10^−11^). Overall, our analyses show that bursts are different between the two cell types, being intrinsically faster and stronger in dominantly bursting cells than in sparsely bursting cells.

### Sparsely and dominantly bursting cells do not segregate on an anatomical level

So far, our analysis has shown that subicular cells could be clustered into two distinct populations with distinct bursting behavior and distinct burst features. In our analysis, we managed to morphologically identify 16 sparsely bursting cells (Fig. 4*A–C*) and 3 dominantly bursting cells (Fig. 4*D–F*). As expected from their spike shapes all could be identified as pyramidal neurons, however, our little data does not allow for firm conclusions about potential morphological differences between sparsely bursting and dominantly bursting cells.

Analysis of subicular burstiness *in vitro* suggested that regular spiking and intrinsic bursting pyramidal cells were not uniformly distributed along the proximodistal in the subiculum, with more bursting cells on the distal part (Greene and Totterdell, 1997; Staff et al., 2000; E. Harris et al., 2001; Y. Kim and Spruston, 2012). A recent study even suggested a sharp transition rather than a gradual evolution of bursting along the proximodistal axis (Cembrowski et al., 2018). These elements led us to check the distribution of sparsely bursting cells and dominantly bursting cells within the subiculum. We could determine the proximodistal level of 94 neurons (proximal, intermediate, or distal) as well as the depth of 30 neurons (superficial, intermediate, or deep). Our analysis showed that dominantly bursting and sparsely bursting cells could be found everywhere within the proximodistal and depth levels of the subiculum. Although dominantly bursting cells seemed to be distributed more toward distal and in deep subiculum, these trends were not significantly different from random distributions (multiple Fisher's exact tests with corrected *p* values; see Materials and Methods; Fig. 4*G–H*). To conclude, it seems the two populations of neurons, well defined by their bursting features, do not show clear anatomical differences.

### Sparsely bursting cells show a stronger spatial modulation than dominantly bursting cells

It was found that cells with distinct bursting behavior could have distinct functions because they tend to project toward different areas (Y. Kim and Spruston, 2012; Cembrowski et al., 2018). For example, cells that project to areas involved in spatial navigation, such as the medial entorhinal cortex or presubiculum, tend to be intrinsic bursting cells when recorded *in vitro*. Therefore, we asked whether sparsely bursting cells and dominantly bursting cells differ in their spatial tuning properties.

We determined the spatial tuning of subicular cells only for recordings where at least 60% of the open-field arena was explored (*n* = 84 of 102). Animals' running trajectories with the superimposed spike positions (Fig. 5*A–C*, left) and the resulting rate maps (Fig. 5*A–C*, middle) were used to calculate each cell's spatial information (see Materials and Methods). Significance of the spatial information was determined using a cell-by-cell circular shuffling procedure. For a given cell, a random and consistent shift in the spike timestamp maintains the temporal structure of firing but alters the spike positions on the rat's trajectory, resulting in a new rate map and different spatial information. Applying this procedure 1000 times allowed us to generate a distribution of spatial information that could be obtained by chance with that cell's firing structure. A cell was then categorized as spatially modulated if its spatial information exceeded the 95th percentile of the distribution obtained by circular shuffling (Fig. 5*A–C*, right).

Figure 5*A* is an example of a sparsely bursting cell. The spikes are mostly confined to the south section of the environment (Fig. 5*A*, left), and the rate map reveals a primary field in the southwest corner and a secondary field (lower rate) in the southeast corner (Fig. 5*A*, middle). Here, the spatial information is 0.89 bits per spike and highly significant (*p* = 0.002), because that value is far above the 95th percentile of the shuffled distribution (Fig. 5*A*, right).

Another sparsely bursting cell is shown in Figure 5*B*. Here, the spike distribution pattern is not as clearly defined, however the rate map shows a clear field all along the southern wall (Fig. 5*B*, middle). This cell also encoded significant spatial information (0.36 bits/spikes; *p* = 0.001; Fig. 5*B*, right) and was categorized as a boundary cell with a significant border score of 0.73 (*p* = 0.012; see Materials and Methods).

A dominantly bursting cell is shown in Figure 5*C*; neither the spikes plotted on the rat's trajectory (Fig. 5*C*, left) nor the rate map (Fig. 5*C*, middle) suggests a sharp spatial tuning. The basal firing rate is very high and spatial information is low (0.06 bits per spikes), but significant (*p* = 0.03), as shown in the comparison with the shuffle distribution (Fig. 5*C*, right).

In total 51 of 84 (63%) subicular cells, including 46 of 69 (67%) sparsely bursting cells and 5 of 15 (33%) dominantly bursting cells were spatially modulated (Fig. 5*D*). Sparsely bursting cells appear to be spatially modulated more often than dominantly bursting cells, as shown by the nonrandom distribution of spatial neurons within the two populations (Fisher's exact test, *p* = 0.022). Looking at the proportion of sparsely bursting cells and dominantly bursting cells within spatially modulated cells (Fig. 5*E*, left), it is clear that sparsely bursting cells constitute a prominent spatial unit within the subiculum. Lastly, 17 of 51 spatially modulated cells (31%) could be classified as boundary cells (as the cell in Fig. 5*B*), and all of them were sparsely bursting cells (Fig. 5*E*, right).

In addition to being less often spatially modulated, spatially tuned dominantly bursting cells provide lower spatial information than sparsely bursting cells (compare Figs. 5*A*,*B*, 5*C*; Fig. 5*F*; medians: SB = 0.36 bits/spike, *n* = 46; DB = 0.16 bits/spike, *n* = 5; *p* = 0.032, tested with bootstrapping procedure; see Materials and Methods). Observing spatial information encoded by the entire population, including cells which are not significantly modulated (Fig. 5*G*), also showed a significant difference between the groups (median: SB = 0.19 bits/spike, *n* = 69; DB = 0.06 bits/spike, *n* = 15; *p* = 0.001, tested with bootstrapping procedure).

To conclude, these last results suggest that our classification of subicular neurons according to burst discharge patterns relates to significant differences in spatial coding.

### Bursts provide spatial information in spatially modulated cells

Here, we classified cells based on each cell's probability to initiate bursts, the least bursting cells being the more spatially modulated. But how is burst firing itself relevant for spatial coding, especially in cells that do not fire bursts often such as sparsely bursting cells? This prompted us to consider how bursts contributed to the transmission of spatial information.

**Figure 6.**
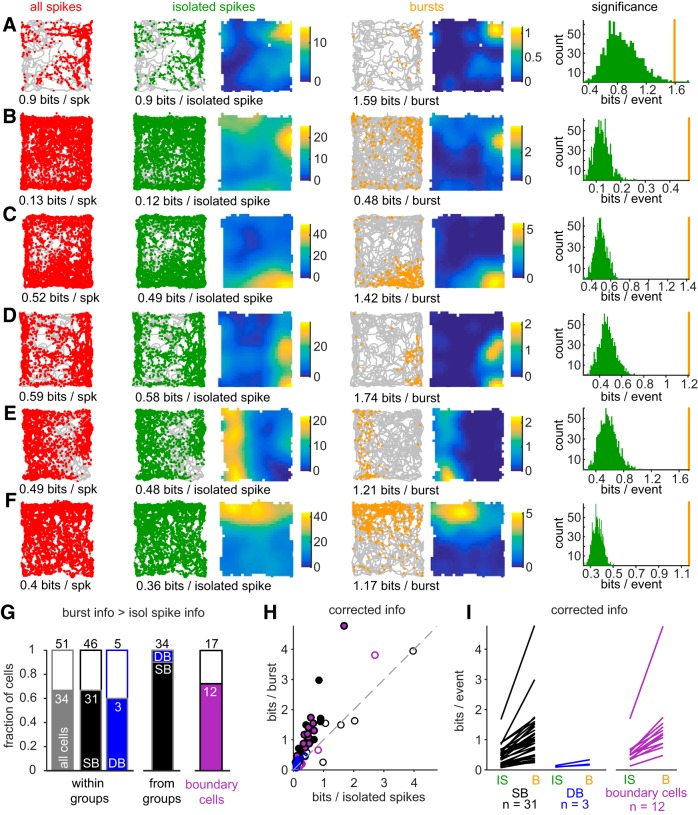
Sharp spatial tuning of burst firing in spatially modulated sparsely bursting cells. ***A–F***, Example plots of sparsely bursting spatial neurons. *Spikes*, Spikes (red dots) superimposed onto the animal's trajectory (gray line). *Isolated spikes*, Isolated spikes (green dots) superimposed onto the animal's trajectory (gray line) and corresponding rate map; corrected spatial information below. *Bursts*, Bursts (orange dots) on animals' trajectories (gray line) and corresponding burst rate maps; spatial information below. Each color-map ranges from 0 to the rate map maximum rate (Hz). *Significance*, Results of the bootstrapping used to determine significance of burst spatial information compared with isolated spike information. The orange line represents spatial information calculated from the *N* bursts fired by each cell and the green histogram shows the distribution of spatial information calculated from 1000 random samples of *N* isolated spikes. Cells in ***C***–***F*** are boundary cells. ***G***, Fraction of cells where burst spatial information is significantly higher than isolated spike spatial information. *Within groups*, Proportion of all spatial cells, sparsely bursting spatial cells and dominantly bursting spatial cells with a significant difference. *From groups*, Proportion sparsely bursting cells and dominantly bursting cells in cells showing a significant difference. *Boundary cells*, Proportion of boundary cells with a significant difference. ***H***, Information per burst versus information per isolated spike for spatially modulated neurons (*n* = 46 sparsely bursting cells, black circles and purple circles corresponding to boundary cells; *n* = 5 dominantly bursting cells, blue circle). Solid circles indicate cells with a significant increase of spatial information between bursts and isolated spikes (*n* = 31/46 for sparsely bursting cells including *n* = 12/17 boundary cells and *n* = 3/5 for dominantly bursting cells). ***I***, Spatial information per isolated spike and bursts only for spatial cells with a significant difference, with sparsely bursting cells in black and dominantly bursting cells in blue and boundary cells in purple. *Corrected info*, Spatial information calculated using similar numbers of bursts and isolated spikes (from the bootstrapping).

The examples shown in Figure 5*A–C* show that many spikes occurred outside of the main spatial firing field. To evaluate how the firing patterns contributed to the coding of spatial information at the single cell level, we separated isolated spikes and bursts into distinct plots. These examples from sparsely bursting cells (Fig. 6*A–F*) suggest that isolated spikes occurred in numerous locations, even though a preferred location is still evident in the rate map (Fig. 6*A–F*, isolated spikes). A strikingly different picture emerged when we only plotted bursts. Well defined firing fields emerged by looking at the bursts' positions on the trajectory and the corresponding rate map (Fig. 6*A–F*, bursts).

The difference in the number of events per group (bursts or isolated spikes) can bias spatial information values; it tends to be higher while computed on a lower number of events (K. D. Harris et al., 2001). For an individual neuron, there were typically more isolated spikes than bursts, so we compared the spatial information from the *n* bursts with 1000 random subsets of *n* isolated spikes. Information was considered higher in bursts if it exceeded the 95th percentile of the distribution of information calculated from the random subsets of isolated spikes (Fig. 6*A–F*, significance; see Materials and Methods for when there are fewer isolated spikes than bursts). The difference between isolated spikes and bursts was significant for 31 of the 46 (67%) spatially modulated sparsely bursting cells and 3 of the 5 (60%) spatially modulated dominantly bursting cells (Fig. 6*G*,*H*). For the 31 sparsely bursting cells with a significant difference, burst spatial information (median = 0.95 bits/burst) was on average 2.31 times higher than isolated spike spatial information (median, 0.41 bits/isolated spike; Fig. 6*I*). Similarly, it was 1.83 times higher for the three dominantly bursting spatial cells, even though the burst spatial information remained among the lowest from our dataset (0.16, 0.19, and 0.33 bits/burst; Fig. 6*I*). Finally, 12 of 17 boundary cells (∼70%; Fig. 6*I*) showed significantly more spatial information in bursts than in isolated spikes. Burst spatial information of boundary cells (median = 1.19 bits/burst) was 2.39 times higher than their isolated spike spatial information (median = 0.5 bits/isolated spike).

## Discussion

We studied how burst firing related to spatial coding in the subiculum of rats. We first classified subicular neurons according to their bursting patterns and distinguished two classes of subicular neurons, a large fraction (80%) of sparsely bursting cells and a small fraction (20%) of dominantly bursting cells. Both cell types are located along the entire proximodistal and radial axes of the subiculum and all identified neurons were pyramidal cells. Most sparsely bursting cells were spatially modulated and we found boundary cells only within that population. Dominantly bursting cells carried little spatial information and were more rarely significantly spatially tuned. Finally, we found that bursts carried more spatial information than isolated spikes in spatially modulated neurons, especially in boundary cells.

The initial impetus for our study came from *in vitro* studies, which identified bursting in subicular neurons. Most interestingly, bursting was shown to be correlated with the projection target of the respective neuron (Y. Kim and Spruston, 2012) suggesting a functional relevance of the bursting phenotype. In previous studies, bursting relationship to spatial coding has been investigated in the subiculum without being clearly defined (Sharp and Green, 1994; Lever et al., 2009; Brotons-Mas et al., 2010; S. M. Kim et al., 2012). Previous reports on CA1 place cells suggested than intrinsic bursting cells, rather than regular spiking cells, were more likely to be spatially modulated (Epsztein et al., 2011). Unlike previous studies on the subiculum, we observed marked functional differences between cell classes defined by their bursting behavior. However, our results were quite opposite to those reported for CA1. Indeed, we found that dominantly bursting cells fire at higher rates and their spikes carry little spatial information, which greatly supported the idea that the overall bursting properties of subicular cells correlates with their spatial coding capabilities. We believe that the high resolution of the juxtacellular recordings, as well as our method to cluster subicular cells in distinct groups reflecting burstiness, was a key element in our findings.

However, it is not yet clear how our *in vivo* classification of sparsely bursting and dominantly bursting cells is related to various classifications of bursting based solely on intrinsic properties. Different *in vitro* studies on subicular neurons reported varying estimates for the fraction of intrinsically bursting neurons, ranging between 45 and 80% (Mason, 1993; Stewart and Wong, 1993; Behr et al., 1996; Greene and Totterdell, 1997; Staff et al., 2000; E. Harris et al., 2001; Y. Kim and Spruston, 2012; Joksimovic et al., 2017). Only ∼20% of the neurons observed in our study were of the dominantly bursting subtype. These numbers do not match previous reports and it seems unlikely that the dominantly bursting cells observed here correspond to the broad definition of intrinsic bursting cells used in *in vitro* studies. It seems possible that the dominantly bursting cells observed by us correspond to a subgroup of neurons with a particularly strong tendency for intrinsic bursting described *in vitro*. In contrast, generating bursts does not appear to be a default mode of firing for most sparsely bursting cells. These cells might be weakly bursting or regular spiking cells requiring more complex mechanisms such as the interaction of intrinsic mechanisms and synaptic inputs for bursting (Larkum, 2013).

A commonly accepted idea is that intrinsic bursting and regular spiking cells, recorded in the slice preparation, are non-uniformly distributed along the proximodistal and radial axes of the subiculum (Greene and Totterdell, 1997; E. Harris et al., 2001; Y. Kim and Spruston, 2012; Cembrowski et al., 2018). Here, we could not observe distinct anatomical distribution of sparsely bursting cells and dominantly bursting cells. Nevertheless, the fact that dominantly bursting cells can be found all along the proximodistal axis of the subiculum challenges the idea that intrinsic bursting cells are found exclusively on the distal portion of the subiculum (Cembrowski et al., 2018). The absence of a clear pattern in our data could be due to the fact that our classification does not identify the classical cell types described *in vitro*. However, as our data suggests that dominantly bursting cells should be a subpopulation of intrinsic bursting cells, it was surprising to find a non-uniform repartition of these cells.

Matching numbers of bursting cells between distinct studies is complicated as experimental conditions may be different from one study to another, especially because burst generation depends on many factors, which are not easy to control *in vivo*. Indeed, a variety of cellular mechanisms of burst generation have been suggested for subicular neurons. For instance, bursting requires T-type voltage gated calcium currents (Joksimovic et al., 2017) that can be affected by neuromodulatory signals, such as serotonin, which was shown to downregulate T-type channels and burst generation in the subiculum (Petersen et al., 2017).

As the output structure of the hippocampus, the subiculum sends high-frequency, but rather imprecise, spatial coding to downstream areas. Indeed, peak frequencies of subicular spatial neurons are rather high compared with CA1, as are their baseline frequencies (Sharp, 1997, 2006; Y. Kim and Spruston, 2012). Nevertheless, spatial signals can be refined if the precise firing pattern of subicular neurons is taken into consideration. Indeed, isolated spikes and bursts are functionally distinct units of information in most spatially modulated neurons (∼60%), especially in cells encoding environmental boundaries. Although bursts were often fired in well defined “place” fields, isolated spikes were more spatially dispersed. Such differential coding by isolated spikes and bursts is similar to information processing in sensory systems (Krahe and Gabbiani, 2004). Nonetheless, our findings are remarkably different from CA1, where bursts sharpen spatial information in only ∼20% of place cells (K. D. Harris et al., 2001). Such a difference shows the relevance of burst firing in noisy spatial cells, such as subicular cells, compared with sharply tuned CA1 place cells.

Bursts and isolated spikes are two units of information that can be read differentially through the interaction between short-term plasticity and postsynaptic integrative properties (Lisman, 1997; Izhikevich et al., 2003). The spatial information conveyed by a burst could be decoded by the summation of excitatory events at facilitating synapses whereas poorly tuned spatial inputs could be better decoded through depressing synapses (Lisman, 1997). This should be the case for long-range projections and could as well define functional subcircuits within the local microcircuit (Simonnet et al., 2017; Nassar et al., 2018). The ongoing activity and resonating properties of targeted neurons could define the response to these signals (Izhikevich et al., 2003). However, the neuronal targets of subicular spatial neurons and how these integrate and convert multiplexed signals at the cellular and microcircuit levels are unknown elements. These will need to be resolved for a better understanding of the subicular role in distributing hippocampal output spatial codes.
